# Application of the Taguchi method to explore a robust condition of tumor-treating field treatment

**DOI:** 10.1371/journal.pone.0262133

**Published:** 2022-01-21

**Authors:** Kosaku Kurata, Kazuki Shimada, Hiroshi Takamatsu

**Affiliations:** 1 Department of Mechanical Engineering, Kyushu University, Fukuoka, Japan; 2 Graduate School of Engineering, Kyushu University, Fukuoka, Japan; Consiglio Nazionale delle Ricerche, ITALY

## Abstract

Tumor-treating fields have potential as minimally invasive cancer treatment. This study aimed to explore the optimum tumor-treating field conditions that minimize unpredicted variations in therapeutic outcomes resulting from differences in cell size and electrical properties. The electric field concentration that induces a dielectrophoretic force near the division plane of a mitotic cell was calculated by finite element analysis for 144 cases, based on different combinations of six noise factors associated with cells and four controllable factors including frequency, as determined by the Taguchi method. Changing the frequency from 200 to 400 kHz strongly increased robustness in producing a dielectrophoretic force, irrespective of noise factors. However, this frequency change reduced the force magnitude, which can be increased by simply applying a higher voltage. Based on additional simulations that considered this trade-off effect, a frequency of 300 kHz is recommended for a robust TTF treatment with allowable variations. The dielectrophoretic force was almost independent of the angle of applied electric field deviated from the most effective direction by ±20 degrees. Furthermore, increased robustness was observed for extracellular fluid with higher conductivity and permittivity. The Taguchi method was useful for identifying robust tumor-treating field therapy conditions from a considerably small number of replicated simulations.

## Introduction

A tumor-treating field (TTF) is a weak AC electric field of several hundred kHz to 1 MHz that interferes with cell division. Despite the low intensity of these electric fields, at only a few volts per centimeter, both cell and animal experiments have confirmed that TTFs can arrest tumor cell proliferation and promote cell destruction [1], which renders TTF as a minimally invasive cancer treatment. A pilot clinical trial targeting recurrent glioblastoma (GBM) demonstrated that an AC electric field of 1–2 V/cm at a frequency of 100–200 kHz nearly doubled both the time to disease progression and overall survival (OS) among those reported thus far [2]. A phase III randomized clinical trial for recurrent GBM, which was conducted following these promising preclinical studies, has demonstrated that TTF treatment results are comparable to those for chemotherapy [3]. It has also been reported that TTF is more effective when combined with chemotherapy [4,5]. Thus, TTF treatment has received the United States Food and Drug Administration (FDA) approval for application to recurrent GBM and newly diagnosed GBM. Following these successes for GBM, numerous trials have been conducted to apply TTF to other cancers in the liver, ovary, lung, and melanoma [6,7]. However, the prognosis for GBM patients is still not satisfactory: the median OS of TTF-treated patients was 14 months, which is only 3 months longer than the OS of those who did not receive TTF treatment [6].

The TTF is considered to impair cell division through several mechanisms. The electric field can modify the alignment of charged molecules, such as tubulin dimers and septin, in a cell. Tubulin dimers are a primary component of microtubules, constituting the spindle for the separation of chromosomes into daughter cells during mitosis. Hence, interference with the tublin dimers due to the force induced by the electric field may disrupt microtubule synthesis and consequently inhibit cell division [8,9]. The electric field also perturbs the localization of septin proteins, which play an important role in protein attachment and barrier formation between mother and daughter cells. Septin dysfunction causes ectopic blebbing and abnormal mitosis [10]. The dielectrophoretic force generated during mitosis may also affect cell division [11]. A dividing cell has an hourglass shape with a cleavage furrow. This shape causes a distortion of the electric field in the cell and results in a concentrated electric field, which generates a dielectrophoretic force on the intracellular polar molecules and thus disrupts normal cell division.

TTF performance is strongly dependent on cell geometry, electrical properties, and the applied electric field. This dependence has been assessed by magnitudes of local intracellular electric fields and dielectrophoretic forces estimated by numerical simulations with assumed electrical properties. The assumed electrical properties of cells were those measured for yeast [12,13], blood cells [14,15], and oocytes [16]. The extracellular electrical properties were assumed to be the same as those of intracellular fluid, blood serum [17,18], or physiological saline [19]. Previous studies have been conducted for combinations of these electric properties, frequency, cell diameter, and membrane thickness (S1 Table) [20–30]. However, because there are many parameters to consider, most of these studies examined the effect of one parameter under a specific combination of other parameters. Therefore, the results highlighted the effects of a major parameter but not the interactions among multiple parameters.

To identify an optimum condition for robust TTF treatment that is not affected by cell geometry or electrical properties, simulations must be conducted for all combinations of parameters for at least six electrical properties: the electrical conductivity and relative permittivity of the intracellular and extracellular fluids and the cell membrane. Such an approach is not realistic because in order to examine three levels for each parameter, for example, simulations must be replicated for 729 (= 3^6^) cases. The number of combinations to be considered can be reduced if we design the analyses using the experimental design method established by R. A. Fisher [31] or, even better, the Taguchi method [32], which is a robust design method originally developed to improve the quality of a product. This method has been recently applied to problems in engineering, biotechnology [33–38], and biomedicine [39–42]. The aim of this study is to investigate an optimum setting for robust TTF therapy. To this end, we performed electrical simulations for a mitotic cell model and conducted an analysis using the Taguchi method to identify a condition that generates a suitable local intracellular electric field and dielectrophoretic force, irrespective of uncontrollable variations in cell geometry and electrical properties.

## Numerical analysis

**Fig 1:**
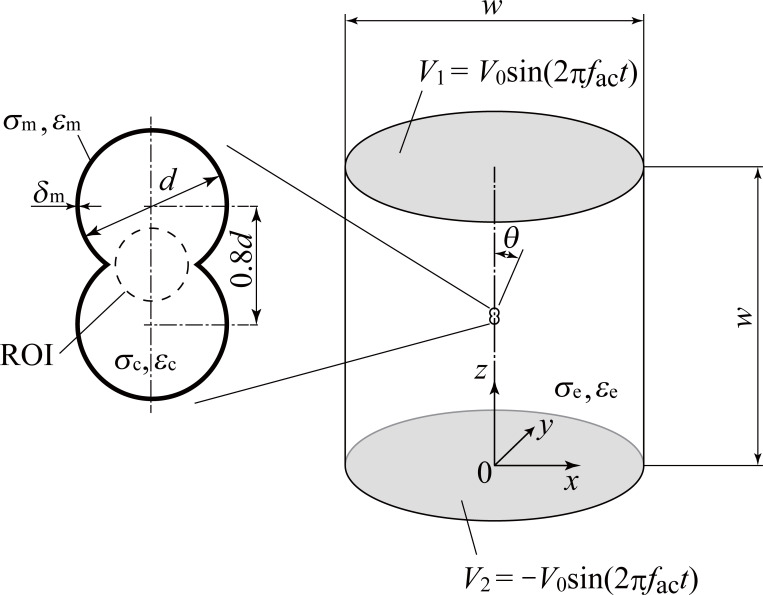
Physical model and coordinate system for calculating the electric field.

We assume a mitotic cell as a target. A three-dimensional cell model was created by overlapping a pair of spherical cells of diameter *d* with a cell membrane thickness of *δ*_m_ and a center-to-center distance of 0.8*d* (Fig 1). To simulate the situation in which the cell is placed in a one-dimensional electric field, we consider a cell in the center of a cylindrical analysis domain that has the same diameter and height *w*, which is sufficiently larger than the cell. The electrical conductivities of the intracellular cytoplasm, cell membrane, and extracellular medium are *σ*_c_, *σ*_m_, *σ*_e_, respectively, and the relative permittivities are *ε*_c_, *ε*_m_, and *ε*_e_, respectively. AC potentials described by

$$V_{1}=V_{0}sin(2\pi f_{ac}t)$$ (1)

$$V_{2}=-V_{0}sin(2\pi f_{ac}t)$$ (2)

are applied to the top and bottom of the analysis domain, respectively, where *V*_0_ is the maximum voltage, *f*_ac_ is the frequency, and *t* is time. The side of the analysis region is treated as electrically insulated.

We consider the charge conservation law:

∇J+∂ρ/∂t=0 (3)

where *J* and *ρ* are the electric current density and charge density, respectively, which are expressed by Ohm’s law and Maxwell’s equation:

J=σE (4)

$$\rho =\nabla ∙(\varepsilon_{0}\varepsilon E)$$ (5)

Herein, *ε*_0_ is the relative permittivity in a vacuum, and *E* is the electric field, defined by the gradient of electric potential *V*:

E=−∇V (6)

By solving these equations via the finite element method, we can determine the distribution of the electric potential and electric field.

If we consider a particle of radius *r*_p_, electrical conductivity *σ*_p_, and relative permittivity *ε*_p_, the dielectrophoretic force *F*_dp_ acting on the particle is calculated from [43,44]:

$$F_{dp}=2\pi r_{p}^{3}\varepsilon_{0}\varepsilon_{c}Re\left(\frac{\varepsilon_{p}^{*}-\varepsilon_{m}^{*}}{\varepsilon_{p}^{*}+{2\varepsilon }_{m}^{*}}\right)\nabla {\left(E_{rms}\right)}^{2}$$ (7)

where *E*_rms_ ($=E/\sqrt{2}$) is the effective electric field and Re indicates the real part of a complex number given in terms of

$$\varepsilon_{p}^{*}=\varepsilon_{p}-i\sigma_{p}/\left(2\pi f_{ac}\right)$$ (8)

$$\varepsilon_{m}^{*}=\varepsilon_{m}-i\sigma_{m}/\left(2\pi f_{ac}\right)$$ (9)

with *i* as the imaginary unit. The term $2\pi r_{p}^{3}\varepsilon_{0}\varepsilon_{c}Re\left(\frac{\varepsilon_{p}^{*}-\varepsilon_{m}^{*}}{\varepsilon_{p}^{*}+{2\varepsilon }_{m}^{*}}\right)$ in (7) is almost constant for a tubulin dimer, a main target of TTF, for an AC electric field on the order of several hundred kilohertz. Thus,

$$\left|F_{dp}|\propto |\nabla {\left(E_{rms}\right)}^{2}\right|$$ (10)

Therefore, we will focus on ∇(*E*_rms_)^2^ to evaluate the magnitude of the dielectrophoretic force.

Finite element analysis was conducted using COMSOL Multiphysics software (v. 5.3a, COMSOL AB, Stockholm, Sweden) equipped with an AC/DC module. Assuming *w* = 20 mm, the cylindrical analysis domain was divided into approximately 180 million tetrahedral elements. Based on previous studies that indicated a successful TTF treatment using intracellular electric fields ranging from 1 to 3 V/cm, we conducted simulations for *V*_0_ = 1, 2, 3, and 4 V, which generate external electric fields of 1, 2, 3, and 4 V/cm, respectively. The simulations were based on various combinations of frequency, cell size, and electrical properties, as summarized in Tables 1 and 2. The effect of a cell membrane that is extremely thin compared with the cell size was considered by applying the contact impedance boundary condition available in COMSOL.

**Table 1:** Controllable factors and levels examined by the Taguchi method.

Controllable factor	Parameters	Level 1	Level 2	Level 3
*C*_1_	Frequency, *f*_ac_ (kHz)	100	200	400
*C*_2_	Direction of cell division, *θ* (deg)	10	0	20
*C*_3_	Electrical conductivity of the extracellular medium, *σ*_e_ (S/m)	0.3	1.2	3
*C*_4_	Relative permittivity of the extracellular medium, *ε*_e_ (-)	60	72.3	80

**Table 2:** Noise factors and levels examined by the Taguchi method.

Noise factor	Parameters	Level 1	Level 2	Level 3
*N*_1_	Thickness of the cell membrane, *δ*_m_ (nm)	3	5	7
*N*_2_	Cell diameter, *d* (μm)	10	20	100
*N*_3_	Electrical conductivity of the cytoplasm, *σ*_c_ (S/m)	0.1	0.3	1
*N*_4_	Relative permittivity of the cytoplasm, *ε*_c_ (-)	60	72.3	80
*N*_5_	Electrical conductivity of the cell membrane, *σ*_m_ (S/m)	1×10^−8^	3×10^−7^	1×10^−6^
*N*_6_	Relative permittivity of the cell membrane, *ε*_m_ (-)	2.5	5	10

**Fig 2:**
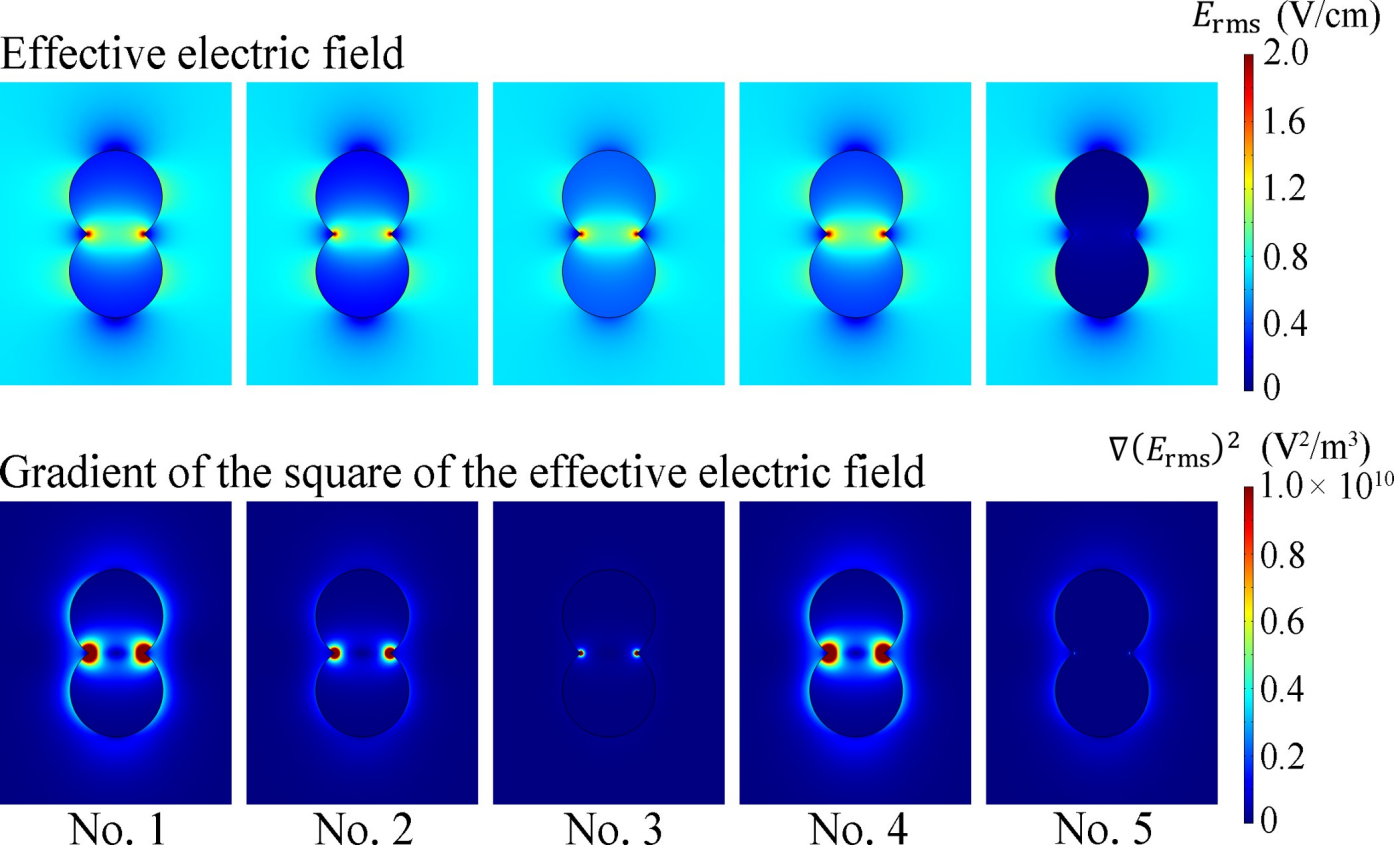
Distribution of the effective electric field *E*_rms_ (top) and the gradient of the square of the effective electric field ∇(*E*_rms_)^2^ (bottom) for *V*_0_ = 1 V, obtained using the parameters classified as level 2 in Table 2 with the other parameters grouped by analysis number from No. 1 to No. 5 in Table 3.

Fig 2 shows examples of the distribution of the effective electric field *E*_rms_ and the gradient of the square of the effective electric field ∇(*E*_rms_)^2^, which were obtained for *V*_0_ = 1 V using the parameters classified as level 2 in Table 1. Five pairs of results correspond to combinations of six other parameters (noise factors described in the next section) grouped by analysis number from No. 1 to No. 5 in Table 3. For case No. 1 to No. 4, a concentration of the electric field was observed at the cleavage furrow of the cell. This concentration resulted in an extremely high ∇(*E*_rms_)^2^ at the neck. In contrast, no increase or concentration of the electric field or ∇(*E*_rms_)^2^ was observed for No. 5. These results indicate that the distribution of ∇(*E*_rms_)^2^ and thus the dielectrophoretic force strongly depend on the electrical properties. Because the results demonstrated that an applied voltage caused the electric field to be concentrated near the cleavage furrow, we defined a spherical region in the center shown in Fig 1 as the region of interest (ROI) and calculated the mean value of ∇(*E*_rms_)^2^. The diameter of the ROI was determined as 80% of the length at the furrow.

**Table 3:** Layout of L18 orthogonal array for noise compounding.

	Noise factor										*δ*_m_ (nm)	*d* (μm)	*σ*_c_ (S/m)	*ε*_c_ (-)	*σ*_m_ (S/m)	*ε*_m_ (-)
Anal. No.	*N*_A_	*N*_B_	*N*_1_	*N*_2_	*N*_3_	*N*_4_	*N*_5_	*N*_6_	*N*_A_	*N*_B_	*N*_1_	*N*_2_	*N*_3_	*N*_4_	*N*_5_	*N*_6_
1	1	1	1	1	1	1	1	1	N/A	N/A	3	10	0.1	60	1×10^−8^	2.5
2	1	1	2	2	2	2	2	2	N/A	N/A	5	20	0.3	72.3	3×10^−7^	5
3	1	1	3	3	3	3	3	3	N/A	N/A	7	100	1	80	1×10^−6^	10
4	1	2	1	1	2	2	3	3	N/A	N/A	3	10	0.3	72.3	1×10^−6^	10
5	1	2	2	2	3	3	1	1	N/A	N/A	5	20	1	80	1×10^−8^	2.5
6	1	2	3	3	1	1	2	2	N/A	N/A	7	100	0.1	60	3×10^−7^	5
7	1	3	1	2	1	3	2	3	N/A	N/A	3	20	0.1	80	3×10^−7^	10
8	1	3	2	3	2	1	3	1	N/A	N/A	5	100	0.3	60	1×10^−6^	2.5
9	1	3	3	1	3	2	1	2	N/A	N/A	7	10	1	72.3	1×10^−8^	5
10	2	1	1	3	3	2	2	1	N/A	N/A	3	100	1	72.3	3×10^−7^	2.5
11	2	1	2	1	1	3	3	2	N/A	N/A	5	10	0.1	80	1×10^−6^	5
12	2	1	3	2	2	1	1	3	N/A	N/A	7	20	0.3	60	1×10^−8^	10
13	2	2	1	2	3	1	3	2	N/A	N/A	3	20	1	60	1×10^−6^	5
14	2	2	2	3	1	2	1	3	N/A	N/A	5	100	0.1	72.3	1×10^−8^	10
15	2	2	3	1	2	3	2	1	N/A	N/A	7	10	0.3	80	3×10^−7^	2.5
16	2	3	1	3	2	3	1	2	N/A	N/A	3	100	0.3	80	1×10^−8^	5
17	2	3	2	1	3	1	2	3	N/A	N/A	5	10	1	60	3×10^−7^	10
18	2	3	3	2	1	2	3	1	N/A	N/A	7	20	0.1	72.3	1×10^−6^	2.5

*N*_A_ and *N*_B_ denote blank columns with no noise factors assigned.

## Taguchi method

Among numerous factors that influence the outcome, those that are controlled by operation are separated from uncontrollable factors that are unknown or that exhibit variations. The Taguchi method was introduced to examine the influence of the controllable factors that display a more robust effect on the outcome irrespective of the uncontrollable factors, which are considered as noise. To this end, the output of interest, ∇(*E*_rms_)^2^ in the ROI, was evaluated for combinations of the controllable factors and noise factors. The signal-to-noise (S/N) ratio, defined as the variation in output with respect to noise, is an important measure for judging the robustness of the influence of controllable factors. Therefore, we sought to identify a combination of controllable factors that maximizes the S/N ratio in order to determine the optimum operation condition that minimizes the effect of noise.

### Parameter diagram of the TTF system

The Taguchi method addresses the optimization of a problem that has a linear relationship between an input *M* and an output *y*, i.e., *y* = *βM*, where *M* is the external electric field in the present study. Based on our preliminary analysis, which confirmed this linear relationship, the square root of the mean ∇(*E*_rms_)^2^ in the ROI is taken as *y*. The proportional constant *β* is determined from simulations conducted for four levels of the input, *M* = 1, 2, 3, and 4 V/cm, and was utilized to evaluate the significance of the electric field.

Four factors, i.e., the frequency of the electric field, the electric field direction with respect to the cell, and the electrical conductivity and relative permittivity of the extracellular medium, were selected as controllable factors (respectively denoted by *C*_*i*_ (*i* = 1−4) in Table 1). The frequency is a major factor that is selected in TTF treatment. The direction of cell division with respect to the external electric field is expected to strongly influence the treatment outcome because the electric field concentration at the neck of the cell division plane is most effective for generating a dielectrophoretic force. Although the relative direction cannot be controlled in treatment owing to the unpredictable and varying directions of cell division, it is considered as a controllable factor to clarify its effect. Similarly, although the electrical conductivity and relative permittivity of the extracellular fluid are not easily controlled, these parameters are included as controllable factors in order to examine the efficacy of administrating exogenous solutions, such as imaging contrast agents, to modify electrical properties.

The remaining factors that significantly influence the outcome but are uncontrollable are selected as noise factors (denoted as *N*_*i*_ (*i* = 1−6) in Table 2). These factors include the cell membrane thickness, cell diameter, and electrical conductivities and relative permittivities of the cytoplasm and cell membrane. The cell membrane thickness and cell diameter are inherently variable and unpredictable. The electrical properties of the intracellular fluid and cell membrane are also uncontrollable and strongly depend on cell type (S1 Table).

**Fig 3:**
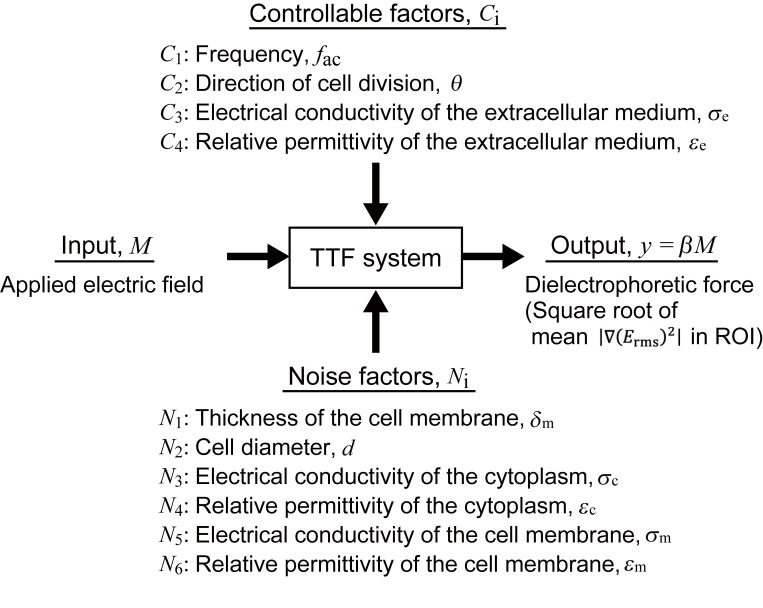
Parameter diagram of the TTF system.

The problem addressed in this work is summarized in Fig 3 as a parameter diagram (P-diagram) that indicates the relationship among the input, output, control factors, and noise factors of the TTF system. The values of the control factors and noise factors, selected for each of three levels, are summarized in Tables 1 and 2. These values were determined based on the parameter ranges shown in S1 Table. The frequency used in current TTF therapeutic devices, the electric field direction considered to maximize the dielectrophoretic force, and the electrical conductivity and relative permittivity values most commonly used in previous studies, i.e., *f*_ac_ = 200 kHz, *θ* = 0, *σ*_e_ = 1.2 S/m, and *ε*_e_ = 72.3, were assigned to level 2, and the combination of level 2 values was denoted as a reference set of controllable factors, *C*_*ref*_.

### Noise compounding

Combinations of the levels of each noise factor used in the analysis are shown in Table 3. Eighteen combinations were selected from an L18 orthogonal array such that the noise factor of each level encountters six times. Using level 2 values for all controllable factors from *C*_1_ to *C*_4_, we conducted numerical analyses for 18 combinations of noise factors for four levels of input *M* to determine $\sqrt{\left|\nabla {\left(E_{rms}\right)}^{2}\right|}$ in the ROI as the output, *y*. The effects of noise factors on *β* and the S/N ratio were evaluated as follows (S2 Table).

The value of *β* is determined by

β=∑My/r (11)

where

$$\sum My=\sum_{i=1}^{4}M_{i}y_{i}$$ (12)

$$r=\sum_{i=1}^{4}{M_{i}}^{2}$$ (13)

The S/N ratio is defined by

$$S/N=10\;log(\beta^{2}/V_{Noise})$$ (14)

where the noise variance, *V*_*Noise*_, is determined from

$$V_{Noise}=S_{Noise}/\left(n-1\right)$$ (15)

based on the following values:

$$S_{T}=\sum_{i=1}^{4}{y_{i}}^{2}$$ (16)

$$S_{\beta }={\left(\sum My\right)}^{2}/r$$ (17)

$$S_{Noise}=S_{T}-S_{\beta }$$ (18)

**Fig 4:**
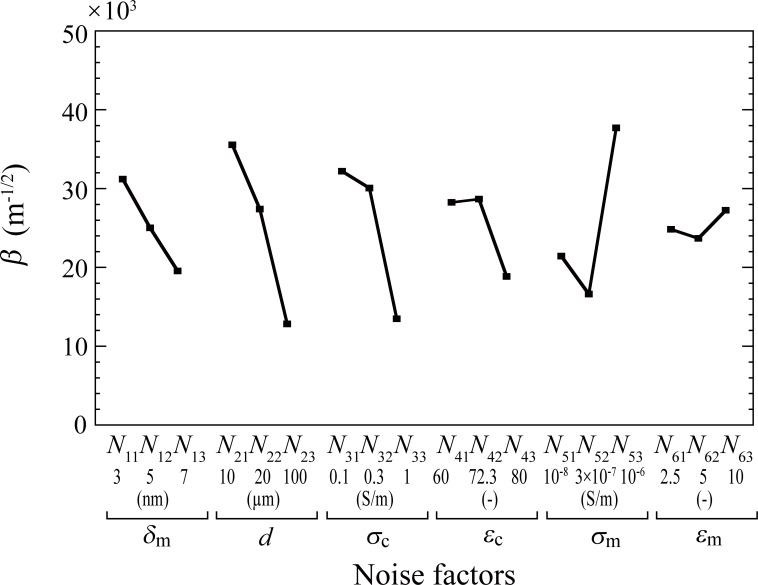
Effect of each noise factor on the mean value of *β*. *N*_ij_ denotes the effect of level *j* for factor *i*.

The value of *β* obtained by averaging that of six data sets for each noise factor, which is denoted by *N*_ij_ for level *j* of noise factor *N*_i_, is shown in Fig 4. Although *β* is lower for higher levels of *N*_1_, *N*_2_, *N*_3_, and *N*_4_, the value for *N*_5_ was the lowest for level 2. The level of *N*_6_ had little effect on *β*. The results shown in Fig 4 indicate that the combination of levels with the highest *β* was *N*_11_−*N*_21_−*N*_31_−*N*_41_−*N*_53_−*N*_63_ whereas the combination with the lowest *β* was *N*_13_−*N*_23_−*N*_33_−*N*_43_−*N*_52_−*N*_62_. Here, we denote these sets of compounded noise factors as *N*_*max*_ and *N*_*min*_, respectively, as summarized in Table 4.

**Table 4:** Combination of noise factors that minimized (*N*_*min*_) and maximized (*N*_*max*_) *β* obtained by noise compounding.

	Compounded noises			
Noise factor	*N*_*min*_		*N*_*max*_	
Thickness of the cell membrane, *δ*_m_ (nm)	*N*_13_	7	*N*_11_	3
Cell diameter, *d* (μm)	*N*_23_	100	*N*_21_	10
Electrical conductivity of the cytoplasm, *σ*_c_ (S/m)	*N*_33_	1	*N*_31_	0.1
Relative permittivity of the cytoplasm, *ε*_c_ (-)	*N*_43_	80	*N*_41_	60
Electrical conductivity of the cell membrane, *σ*_m_ (S/m)	*N*_52_	3×10^−7^	*N*_53_	1×10^−6^
Relative permittivity of the cell membrane, *ε*_m_ (-)	*N*_62_	5	*N*_63_	10

### Factorial effects of control factors

Next, with the set of noise factors fixed at *N*_*max*_ and *N*_*min*_, finite element analyses were conducted for 18 combinations of four controllable factors selected from the L18 orthogonal array, which includes six cases of the same level for each controllable factor (S3 Table). Again, the analyses were performed for four levels of input *M*. The values of *β* and the S/N ratio were determined by Eqs (11) and (14), respectively.

**Fig 5:**
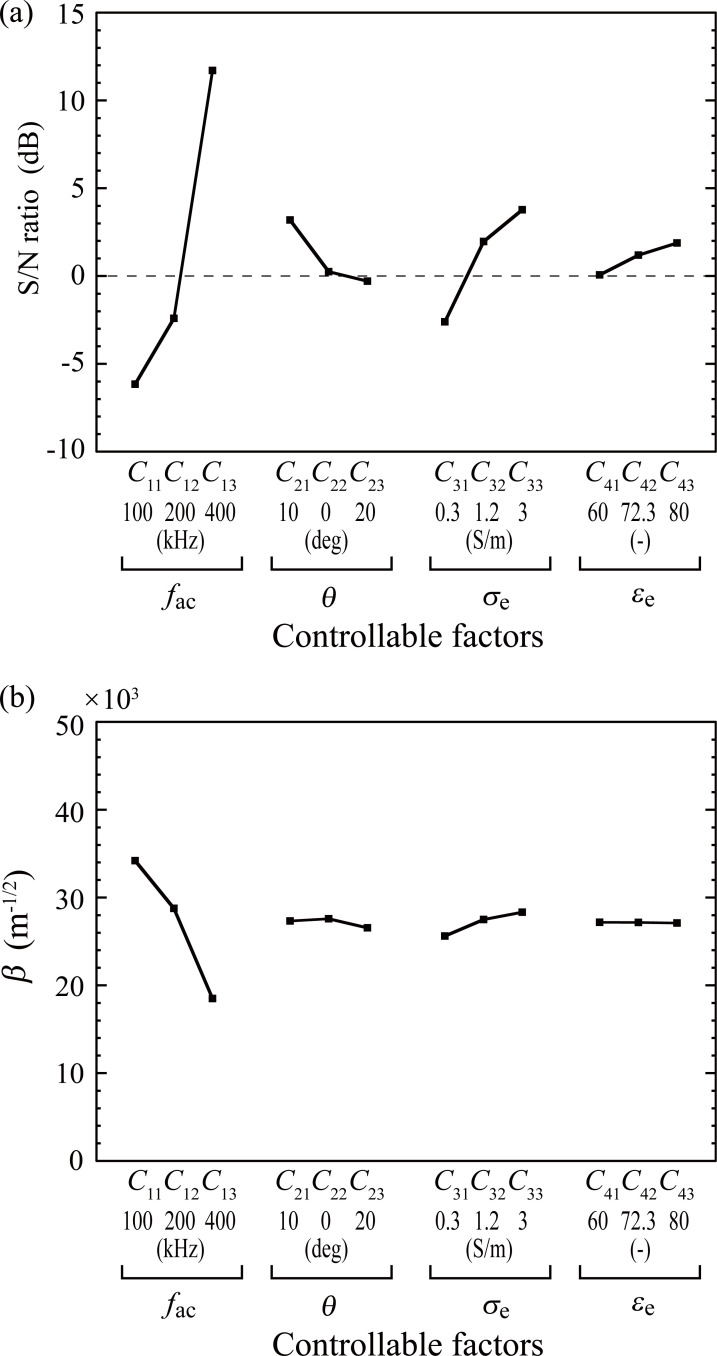
Effect of each controllable factor on the mean of the S/N ratio (a) and *β* (b). *C*_ij_ denotes the effect of level *j* for factor *i*.

Fig 5 shows the effect of controllable factors on *β* and the S/N ratio, with each given as the average of six data sets for each controllable factor *C*_ij_, the level *j* of factor *C*_i_. The influence of factor *C*_1_ (frequency) on both the S/N ratio and *β* was much greater than that of the other factors. Although the average value of *β* was the largest for level 1 (*C*_11_), the S/N ratio was extremely small, suggesting that the output was easily influenced by noise. Therefore, an increased input (applied electric field) did not always result in a considerable increase in the output. In contrast, *C*_13_ showed a very high S/N ratio and a moderate *β*, indicating a solid effect irrespective of the combinations of other factors. Therefore, a robust effect is expected for *C*_13_, level 3 of *C*_1_. For the same reason, the condition that resulted in the highest S/N ratio for each factor is preferable. Thus, we obtained the combination *C*_13_−*C*_21_−*C*_33_−*C*_43_ as an optimum set of controllable factors, *C*_*opt*_.

**Fig 6:**
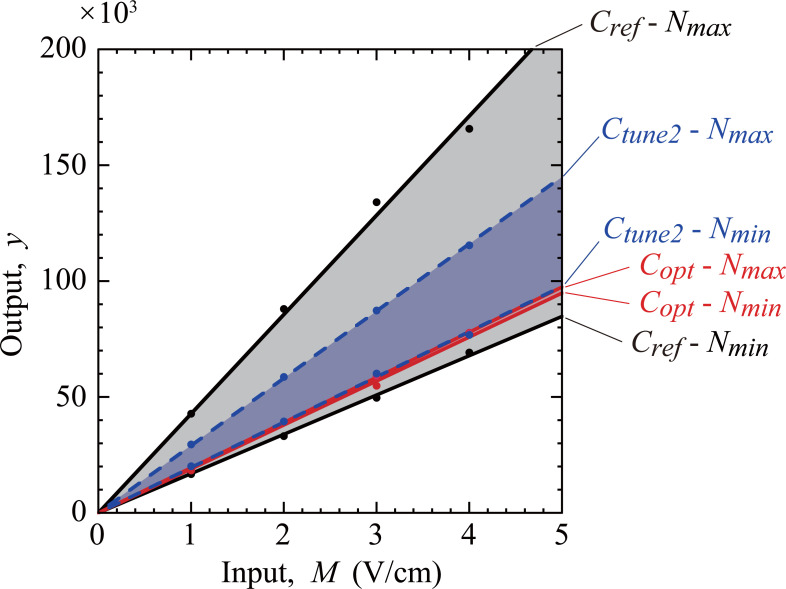
Relationship between the input *M* (applied electric field) and the output *y* (square root of ∇(*E*_rms_)^2^).

The increase in the output *y* ($\sqrt{\left|\nabla {\left(E_{rms}\right)}^{2}\right|}$ in the ROI) with the input *M* (applied electric field) for the above-determined conditions is shown in Fig 6. The output increased almost linearly under all conditions, satisfying the requirement of the Taguchi method. The values of *y* obtained for level 2 of all controllable factors, *C*_*ref*_, with the set of noise factors *N*_*min*_ was the lowest, whereas that with *N*_*max*_ was the highest, being more than two-fold higher than the former result. Hence, the result obtained for the other combinations of noise factors with *C*_*ref*_ will fall between these two lines. In contrast, the result for the set of control factors *C*_*opt*_ and *N*_*max*_ was similar to that for *C*_*opt*_ and *N*_*min*_, with a difference of 3%. This finding suggests that the result for the set of controllable factors *C*_*opt*_ was almost independent of noise factors, indicating that the output was robust.

### Validation and tuning

Although *β* was not influenced by noise factors for the set of controllable factors *C*_*opt*_, *β* was much smaller than the highest value obtained for *C*_*ref*_ and *N*_*max*_. Therefore, the final goal of the analysis is to find the optimum condition, i.e., a combination of controllable factors that results in a higher *β* with an allowable S/N ratio.

First, finite element analysis was conducted for four combinations of controllable factors and noise factors, *C*_*opt*_ (*C*_13_−*C*_21_−*C*_33_−*C*_43_)−*N*_*min*_, *C*_*opt*_−*N*_*max*_, *C*_*ref*_ (*C*_12_−*C*_22_−*C*_32_−*C*_42_)−*N*_*min*_, and *C*_*ref*_−*N*_*max*_ with inputs of *M* = 1, 2, 3, and 4 V/cm. To determine whether the results could be predicted from the data that had already been obtained, the S/N ratio, *η*_opt_, *η*_*ref*_, and the values of *β*, *β*_opt_, and *β*_*ref*_ were predicted from the following equations:

$$\eta_{opt}=\eta_{T}+(\eta_{C13}-\eta_{T})+(\eta_{C21}-\eta_{T})+(\eta_{C33}-\eta_{T})+(\eta_{C43}-\eta_{T})$$ (19)

$$\eta_{ref}=\eta_{T}+(\eta_{C12}-\eta_{T})+(\eta_{C22}-\eta_{T})+(\eta_{C32}-\eta_{T})+(\eta_{C42}-\eta_{T})$$ (20)

$$\beta_{opt}=\beta_{T}+(\beta_{C13}-\beta_{T})+(\beta_{C21}-\beta_{T})+(\beta_{C33}-\beta_{T})+(\beta_{C43}-\beta_{T})$$ (21)

$$\beta_{ref}=\beta_{T}+(\beta_{C12}-\beta_{T})+(\beta_{C22}-\beta_{T})+(\beta_{C32}-\beta_{T})+(\beta_{C42}-\beta_{T})$$ (22)

where *η*_*Cij*_ and *β*_*Cij*_ are the values for level *j* of controllable factor *i*, respectively, and *η*_T_ and *β*_T_ are the average of all data.

The results are listed in Table 5. The numerical simulation showed that as the parameters changed from *C*_*ref*_ to *C*_*opt*_, the S/N ratio increased by 25.2 dB, whereas *β* decreased from 29.9×10^3^ m^−1/2^ to 19.2×10^3^ m^−1/2^. This trend indicates that although *β* decreased by 36%, the output fluctuation decreased considerably to 5.5% of that for *C*_*ref*_. The percentage variation relative to the compared condition, *P* (%), corresponding to an S/N ratio increase of *x* dB (gain Δ) is

$$P=0.5^{\frac{x}{6}}\times 100$$ (23)

**Table 5:** Comparison of the S/N ratio and *β* obtained for different sets of parameters.

		S/N ratio, *η* (dB)					
Combination		Predicted		Gain Δ *η-η*_*ref*_	Validated		Gain Δ *η-η*_*ref*_
*C*_*ref*_	*C*_12_-*C*_22_-*C*_32_-*C*_42_	-2.1			-2.1		
*C*_*opt*_	*C*_13_-*C*_21_-*C*_33_-*C*_43_	17.4		19.6	23.1		25.2
*C*_*tune1*_	*C*_12_-*C*_21_-*C*_33_-*C*_43_	3.3		5.4	-1.8		0.2
*C*_*tune2*_	300kHz-*C*_21_-*C*_33_-*C*_43_	N/A		N/A	4.9		6.9
		*β* (m^-1/2^)					
Combination		Predicted		*β/β*_*ref*_	Validated		*β/β*_*ref*_
*C*_*ref*_	*C*_12_-*C*_22_-*C*_32_-*C*_42_	29.6	×10^3^		29.9	×10^3^	
*C*_*opt*_	*C*_13_-*C*_21_-*C*_33_-*C*_43_	19.8		0.67	19.2		0.64
*C*_*tune1*_	*C*_12_-*C*_21_-*C*_33_-*C*_43_	30.1		1.02	31.2		1.04
*C*_*tune2*_	300kHz-*C*_21_-*C*_33_-*C*_43_	N/A		N/A	24.3		0.81

The increase in the S/N ratio of the predicted result was relatively smaller, 19.6 dB, corresponding to a variation reduction to 10.4% of that for *C*_*ref*_. However, the other values, including *β* for *C*_*ref*_ and *C*_*opt*_ and the S/N ratio for *C*_*ref*_, agreed well with the numerical simulation, indicating the validity of the Taguchi method.

We found that selecting the combination *C*_*opt*_ rather than *C*_*ref*_ considerably increases the robustness, with much less influence due to noise factors. However, *β* was much smaller, suggesting that a higher voltage is needed to induce an electric field that can generate an adequate dielectrophoretic force. Although increasing both *β* and the S/N ratio is not possible because of their trade-off relationship, it is possible to find a condition that results in a higher *β* with an allowable S/N ratio. Because the frequency has a significant effect on the outcome, 200 kHz (*C*_12_) and 300 kHz (not included in Table 1) were examined instead of 400 kHz (*C*_13_) in *C*_*opt*_ by additional numerical simulations. In the result for 200 kHz, the S/N ratio was much lower than that for *C*_*opt*_ and only 0.2 dB higher than that for *C*_*ref*_, even though *β* was considerably higher (Table 5). In contrast, compared with the result for *C*_*opt*_, the application of 300 kHz increased *β* by 27% from 19.2×10^3^ m^−1/2^ to 24.3×10^3^ m^−1/2^ whereas the S/N ratio was maintained at 4.9 dB, which is 18.3 dB lower than that for *C*_*opt*_ but 6.9 dB higher than that for *C*_*ref*_.

## Discussion

In this study, a robust condition of TTF treatment was identified, with the goal of minimizing the variation in therapeutic outcomes irrespective of differences in cell size and electrical properties. TTF therapy aims to generate a dielectrophoretic force on intracellular molecules by application of an electric field, ultimately interfering with cell division. Based on a linear relationship between the applied electric field (input *M*) and the square root of the dielectrophoretic force (output *y*) at the ROI, i.e., *y* = *βM*, we identified a TTF condition that minimizes the variation in *β* owing to unpredicted variations in unknown cell sizes and electrical properties.

We first selected four controllable factors and six uncontrollable noise factors that influence *β*. If three levels of each factor are to be examined, the simulation must be replicated 3^10^ (= 59,049) times for all combinations of the selected factors. Therefore, the Taguchi method, which is a powerful tool for optimization problems, was used to reduce the number of replicates by using an orthogonal array of combination factors. The main effect and interaction of each factor were simultaneously evaluated by combining each level of a factor equally with the other factors in the orthogonal array. Considering that the assignment of four controllable factors and six noise factors to the L18 orthogonal array and four levels of applied electric field (input *M*) still requires a simulation of 1,296 (= 18 × 18 × 4) replicates, the number of simulations was further decreased by compounding noise factors into two combinations that maximize and minimize *β*, respectively, which were determined before the control factors were examined. By assigning four control factors to the L18 orthogonal array with two combinations of noise factors and four levels of applied electric field, the numerical simulation was replicated for 144 (= 18 × 2 × 4) cases.

The six uncontrollable factors considered as noise were the cell membrane thickness, cell diameter, and electrical conductivities and relative permittivities of the intracellular fluid and cell membrane. The membrane thickness was assumed to be 3–7 nm in this study, and the cell diameter was assumed as 10–100 μm based on previous studies (S1 Table). The assumed range of electrical properties covered those used in most of these previous studies. The electrical properties of the intracellular fluid and cell membrane strongly depend on cell type [45,46], and those measured for cultured cells may differ from those in the human body in an aggregate state. Therefore, finding a reliable condition for a variety of these properties is important for successful TTF therapy.

The frequency of the applied electric field was the first-priority controllable factor, which ranged from 100 to 400 kHz. This range, which is much narrower than that employed in previous studies (a few kHz to several GHz (S1 Table)), was selected based on the finding that several hundred kHz is the most effective frequency for inhibiting cell proliferation [11,28]. Actually, frequencies of 200 and 150 kHz are used in the Optune system (Novocure Ltd.) and Novo TTF-100L, respectively, which are commercially available TTF therapeutic devices. We found that a lower frequency can potentially increase the dielectrophoretic force. However, this trend largely depends on other factors, indicating a considerably lower S/N ratio at lower frequencies (Fig 5). This trade-off effect of frequency on the dielectrophoretic force and the S/N ratio is one of the most important findings in this study.

The second controllable factor, the direction of the applied electric field with respect to the plane of cell division, cannot be controlled in clinical TTF therapy owing to the unknown, unpredictable direction of cell division in the human body. It is obvious that the largest dielectrophoretic force is produced when the electric field is applied perpendicular to the division plane, because the dielectrophoretic force is a result of electric field distortion near the cleavage furrow. However, the direction was considered as a controllable factor to clarify how the dielectrophoretic force decreases as the direction deviates from the desired angle. The results indicate that the dielectrophoretic force is nearly independent of the angle within a range of ±20 degrees, showing a variation of less than 4 dB (Fig 5). This finding suggests that using five pairs of electrodes placed at angles of 36 degrees each around a target tissue will produce an adequate dielectrophoretic force in the cells, independent of the direction of cell division. To further investigate the effect of the direction of the electric field on the robustness of TTF, additional analysis was conducted, in which the angles shifted to 20°, 30°, and 45° were assigned to the L18 orthogonal array. The S/N ratio and *β* obtained from this analysis are shown in Table 6. Even if the angle was changed from the most robust condition of *C*_*opt*_ to 20° (*C*_20°_), the S/N ratio was still 18.3 dB higher than that for *C*_*ref*_. This indicates that the output fluctuation decreased to 12.1% of that for *C*_*ref*_. As the angle was further changed to 30° (*C*_30°_) and 45° (*C*_45°_), the improvement in robustness was decreased to 8.2 dB and 2.9 dB, which resulted in the output fluctuation being reduced to only 38.8% and 72.5% of *C*_*ref*_, respectively. On the other hand, the effect of the angle on *β* was negligible; *β* decreased irrespective of the angle, and was approximately 0.7 times as small as that for *C*_*ref*_.

**Table 6:** Comparison of the S/N ratio and *β* obtained for different angles.

		S/N ratio, *η* (dB)					
Combination		Predicted		Gain Δ *η-η*_*ref*_	Validated		Gain Δ *η-η*_*ref*_
*C*_*ref*_	*C*_12_-*C*_22_-*C*_32_-*C*_42_	-2.1			-2.1		
*C*_*opt*_	*C*_13_-*C*_21_-*C*_33_-*C*_43_	17.4		19.6	23.1		25.2
*C*_*20°*_	*C*_13_-20°-*C*_33_-*C*_43_	13.9		16.0	16.2		18.3
*C*_*30°*_	*C*_13_-30°-*C*_33_-*C*_43_	N/A		N/A	6.1		8.2
*C*_*45°*_	*C*_13_-45°-*C*_33_-*C*_43_	N/A		N/A	0.8		2.9
		*β* (m^-1/2^)					
Combination		Predicted		*β/β*_*ref*_	Validated		*β/β*_*ref*_
*C*_*ref*_	*C*_12_-*C*_22_-*C*_32_-*C*_42_	29.6	×10^3^		29.9	×10^3^	
*C*_*opt*_	*C*_13_-*C*_21_-*C*_33_-*C*_43_	19.8		0.67	19.2		0.64
*C*_*20°*_	*C*_13_-20°-*C*_33_-*C*_43_	19.0		0.64	19.9		0.67
*C*_*30°*_	*C*_13_-30°-*C*_33_-*C*_43_	N/A		N/A	20.4		0.68
*C*_*45°*_	*C*_13_-45°-*C*_33_-*C*_43_	N/A		N/A	20.5		0.69

**Fig 7:**
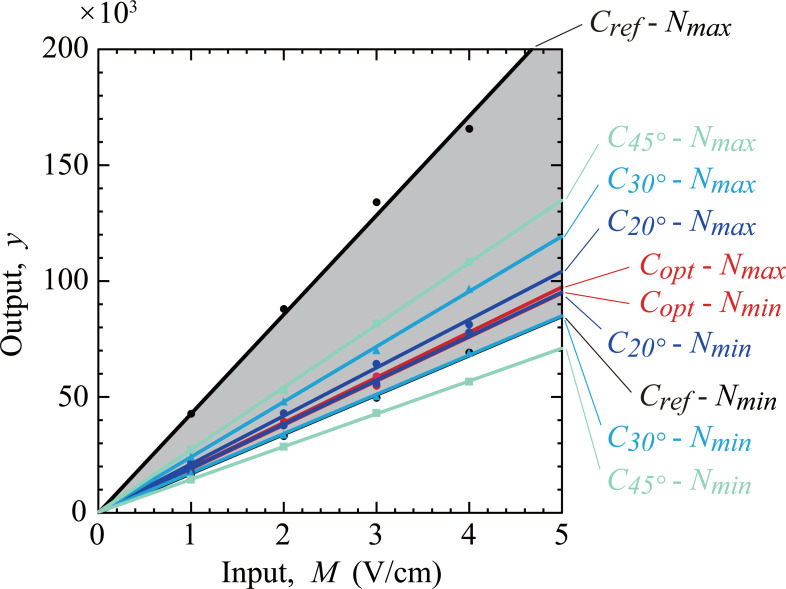
Relationship between the input *M* (applied electric field) and the output *y* (square root of ∇(*E*_rms_)^2^) obtained for different angles.

TTF efficacy is maximal when the axis of cell division is parallel to the electric field [1,2]. Therefore, in clinical applications, TTF is commonly delivered to a target tumor by using multiple pairs of electrodes because cells undergo mitosis in different spatial orientations [47,48]. The analysis for *θ* = 20°, 30°, and 45° corresponds to the treatment using five pairs, three pairs, and two pairs of electrodes, respectively (S1 Fig). Increasing the number of electrode pairs from two to three and five improved the robustness by 5.3 dB (= 6.1–0.8 dB) and 15.4 dB (= 16.2–0.8 dB), respectively, which indicates that the output fluctuation decreased to 54.2% and 16.9%. Fig 7 shows the increase in the output *y* with the input *M* for angles of 20°, 30°, and 45°. The result for *C*_20°_ (*θ* = 20°) and noise factors *N*_*max*_ was only 9.4% different from that for the set of *C*_20°_ and *N*_*min*_. The difference caused by uncontrollable noises increased to 41% for *C*_30°_ (*θ* = 30°) and 90% for *C*_45°_ (*θ* = 45°). This indicates that TTF treatment using two orthogonal sets of electrodes is much more susceptible to noise factors than that using five sets of electrodes. However, there are limitations to this finding; electrodes on a single plane may not be sufficient to obtain the expected effect because cells will divide in all directions three-dimensionally while being exposed to non-uniform electric fields depending on the location. Furthermore, the feasibility of a multiple-electrode setup would be an issue for clinical applications. In order to take the complex anatomy and unknown direction of cell division into account, it is essential to combine a MR image-based finite element model of tumors with a realistic electrode layout, as previously reported by Korshoej et al. [49–51].

The electrical properties of the extracellular fluid, i.e., the third and fourth controllable factors, are inherently uncontrollable, but were included as controllable factors to examine the feasibility of changing these properties by injecting an exogenous solution. A higher extracellular conductivity increased the robustness of the TTF treatment (Fig 5(A)) and slightly increased the dielectrophoretic force (Fig 5(B)). This increased conductivity can be achieved by injecting saline (1.4 S/m), which has a conductivity slightly higher than that of blood serum (1.2 S/m), considered as level 2 in this study. However, other solutions such as angiographic agents should not be used because of their lower electric conductivity: 0.5 S/m for Gd-based electrolytic contrast agents of magnetic resonance imaging (MRI), such as Magnevist and Dotarem, and 0.01 S/m for a non-electrolytic Fe-based agent, EM1301 [52]. Therefore, the administration of exogenous agents is not desirable for enhancing the TTF effect. More importantly, it should be noted that the MRI contrast agent used for imaging may have a negative effect on the robustness of the treatment if the agent remains in the target area. Increasing another factor, the dielectric effect, also slightly increased the S/N ratio. However, in this respect, no liquid seems to be superior to water, which has a high relative permittivity of 80.

In agreement with a previous study [28], we found that the dielectrophoretic force was significantly smaller in larger cells (Fig 4). A previous study reported unintended cell expansion during TTF treatment [53]. Therefore, it is plausible that repeated TTF therapy will become less effective for cells that become larger due to TTF *per se*.

In this work, the most important indicator for the optimization problem is the S/N ratio, which represents the robustness of the outcome. Even when the output *β* is high on average, it does not mean that the application of a higher voltage always produces large dielectrophoretic forces in cells if the S/N ratio is considerably low. Meanwhile, if the S/N ratio is relatively high, we can induce larger dielectrophoretic forces by applying a higher voltage only if *β* is positive. From this viewpoint, the optimum condition *C*_*opt*_ (*C*_13_−*C*_21_−*C*_33_−*C*_43_) was considerably better than *C*_*ref*_ (*C*_12_−*C*_22_−*C*_32_−*C*_42_) because the S/N ratio was higher by 25.2 dB (Table 5), corresponding to a reduced variation in the output, which decreased to one eighteenth of that for *C*_*ref*_. However, to obtain the same magnitude of output ($\sqrt{\left|\nabla {\left(E_{rms}\right)}^{2}\right|}$) as that for the *C*_*ref*_ condition, the application of an electric field 1.56 (= 1/0.64) times stronger than that for the *C*_*ref*_ condition is needed because *β* is 36% smaller. Hence, the voltage must be 2.4 (= 1.56^2^) times higher to induce the same dielectrophoretic force.

The difference between the results for *C*_*opt*_ and *C*_*ref*_ was primarily due to the opposing effects of frequency on the output and S/N ratio. Considering this trade-off effect, we sought for conditions that yield a higher output with an allowable S/N ratio by changing the frequency. Consequently, we found that a frequency of 300 kHz, which was between level 2 (200 kHz) and level 3 (400 kHz), resulted in a *β* value that was 27% higher than that obtained for *C*_*opt*_ at the expense of a somewhat smaller S/N ratio, which was 18.2 dB lower than that for *C*_*opt*_ (12.2% larger in variation) but 6.9 dB higher (54.9% lower) than that for *C*_*ref*_ (Table 5). Therefore, we recommend a frequency of 300 kHz for robust TTF treatment.

## Conclusion

The optimum condition of TTF treatment with a high robustness to unpredictable variations in cell size and electrical properties was investigated by numerical simulations in which an AC voltage was applied to a mitotic cell. The magnitude of the dielectrophoretic force near the plane of cell division was evaluated for different voltages for combinations of six uncontrollable noise factors (cell membrane thickness, cell diameter, and electrical conductivities and relative permittivities of the intracellular fluid and cell membrane) and four controllable factors (frequency, electric field direction, and electrical conductivity and relative permittivity of the extracellular fluid). The number of simulation replicates needed for the combinations of these factors was reduced to 144 cases using the Taguchi method. Increasing the frequency significantly increased the robustness of the dielectrophoretic force irrespective of noise factors but considerably decreased the magnitude of the force. Based on additional simulations that considered this trade-off effect, a frequency of 300 kHz is recommended for a robust TTF treatment with allowable variations. The dielectrophoretic force was almost independent of the angle of the applied electric field for deviations of ±20 degrees from the most effective direction. This study also demonstrated the effect of the electrical properties of the extracellular fluid, suggesting the feasibility of administrating saline as an exogenous solution to change the properties of the extracellular fluid. However, it should be noted that the MRI contrast agent used for imaging may have a negative effect on the TTF outcome.

## Supporting information

S1 Fig Geometric relationship between a cell and electrodes with angles of 20°, 30°, and 45°. (EPS)

S1 Table List of parameters used in previous studies: Frequencies of applied electric field, cell geometry, and electrical properties of the cytoplasm, cell membrane and extracellular medium. (PDF)

S2 Table S/N ratio and *β* calculated by assigning noise factors and input *M* to L18 orthogonal array. (PDF)

S3 Table S/N ratio and *β* calculated by assigning control factors, compounded noise factors, and input *M* to L18 orthogonal array. (PDF)
