# Supplementary material for: Application of the Taguchi method to explore a robust condition of tumor-treating field treatment
Source: PLoS One. 2022 Jan 21;17(1):e0262133. doi: 10.1371/journal.pone.0262133 (PMC8782397; doi:10.1371/journal.pone.0262133)
Supplement: S1 Table — (PDF) [file pone.0262133.s002.pdf]

Frequency of the applied voltage, f_{ac} (Hz)	Thickness of the cell membrane, δ_m (nm)	Cell diameter, d (μm)	Electrical conductivity of the cytoplasm, σ_c (S/m)	Relative permittivity of the cytoplasm, ε_c (-)	Electrical conductivity of the cell membrane, σ_m (S/m)	Relative permittivity of the cell membrane, ε_m (-)	Electrical conductivity of the extracellular medium, σ_e (S/m)	Relative permittivity of the extracellular medium, ε_e (-)	Reference
1k–1G	5	8, 20, 30, 40	0.3	72.3	3×10^{-7}	5	1.2	72.3	C. Wenger, et al., 2018
60–1G	5	20	0.1, 0.3, 0.5, 0.75, 0.9	60, 72.3, 80	3×10^{-7}, 1×10^{-6}, 1×10^{-5}, 5×10^{-5}	2.5, 5, 7.5	1.2	60, 72.3, 80	C. Wenger, et al., 2015
100k–500k	5	20	0.3 (Normal)<br>0.5 (Tumor)	60 (Normal)<br>60 (Tumor)	3×10^{-7} (Normal)<br>5×10^{-6} (Tumor)	12.8 (Normal)<br>9.8 (Tumor)	0.6	80	X. Li, et al., 2020
100k, 13.56M	7	10	0.3	80	3×10^{-7}	3	0.3	80	P. K. Tiwari, et. al., 2009
1–10G	5	20	0.3	72.3	3×10^{-7}	5	1.2	72.3	T. R. Gowrishankar, et. al., 2006
DC	5	2, 20, 200	0.02, 0.2, 1	-	1×10^{-8}, 5×10^{-7}, 1.2×10^{-6}	0.01 F/m^2	0.0005, 0.2, 2	-	T. Kotnik, et. al., 1997
10–1G	5	20	0.3	72.3	3×10^{-7}	5	1.2	72.3	T. Kotnik, et. al., 2000
16k–1.6G	3–7	10–200	0.1–1	39.5–79.1	1×10^{-8}–1×10^{-5}	2–10	0.1–1.0	39.5–79.1	T. Kotnik, et. al., 2006
1–2G	5	20	0.3	72.3	3×10^{-7}	4.98	1.2	72.3	D. Stewart, et. al., 2005
DC	3–7	10–40	0.02–1	-	1×10^{-8}–1.2×10^{-6}	-	0.0005–2	-	H. Ye, et al., 2012
2k–200k	3–7	10–200	0.1–1	39.5–79.1	1×10^{-8}–1×10^{-6}	2–10	1.2	72.3	H. Ye, et al., 2010
