# Supplementary material for: Application of the Taguchi method to explore a robust condition of tumor-treating field treatment
Source: PLoS One. 2022 Jan 21;17(1):e0262133. doi: 10.1371/journal.pone.0262133 (PMC8782397; doi:10.1371/journal.pone.0262133)
Supplement: S2 Table — (PDF) [file pone.0262133.s003.pdf]

	Noise factor										Thickness of the cell membrane, δ_m (nm)	Cell diameter, d (μm)	Electrical conductivity of the cytoplasm, σ_c (S/m)	Relative permittivity of the cytoplasm, ε_c (-)	Electrical conductivity of the cell membrane, σ_m (S/m)	Relative permittivity of the cell membrane, ε_m (-)	Output: Square root of mean $\lvert\nabla(E_{rms})^2\rvert$ in ROI				Sum of squared inputs	Sum of products of input and output	Sum of squared outputs (Total variation)	Variation of proportional term	Variation of noise	Variance of noise	Slope	S/N ratio $S/N = \eta_{db}$ $= 10log\frac{\beta^2}{\sigma_{Noise}^2}$ $= 10log\frac{\beta^2}{V_{Noise}}$
Anal. No.	N_A	N_B	N_1	N_2	N_3	N_4	N_5	N_6	N_A	N_B	N_1	N_2	N_3	N_4	N_5	N_6	Input (V/cm): M_1 1	M_2 2	M_3 3	M_4 4	$r = \sum_{i=1}^{4} M_i^2$	$\sum My = \sum_{i=1}^{4} M_i y_i$	$S_T = \sum_{i=1}^{4} y_i^2$	$S_\beta = \frac{1}{r}\left(\sum My\right)^2$	$S_{Noise} = S_T - S_\beta$	$\sigma_{Noise}^2 = V_{Noise} = \frac{S_{Noise}}{n-1}$	$\beta = \frac{1}{r}\sum My$	
1	1	1	1	1	1	1	1	1	N/A	N/A	3	10	0.1	60	1×10^{-8}	2.5	62.4×10^{3}	123×10^{3}	185×10^{3}	246×10^{3}	30	1.85×10^{6}	114×10^{9}	114×10^{9}	1079×10^{3}	360×10^{3}	61.6×10^{3}	40.2
2	1	1	2	2	2	2	2	2	N/A	N/A	5	20	0.3	72.3	3×10^{-7}	5	39.7	79.0	118	158	30	1.19	46.9	46.9	56.6	18.9	39.5	49.2
3	1	1	3	3	3	3	3	3	N/A	N/A	7	100	1	80	1×10^{-6}	10	17.7	35.6	53.2	71.2	30	0.533	9.49	9.49	22.5	7.48	17.8	46.3
4	1	2	1	1	2	2	3	3	N/A	N/A	3	10	0.3	72.3	1×10^{-6}	10	63.1	126	189	252	30	1.89	119	119	36.6	12.2	63.0	55.1
5	1	2	2	2	3	3	1	1	N/A	N/A	5	20	1	80	1×10^{-8}	2.5	3.48	6.92	10.4	14.5	30	0.107	0.379	0.379	174	58.2	3.55	23.4
6	1	2	3	3	1	1	2	2	N/A	N/A	7	100	0.1	60	3×10^{-7}	5	5.61	11.3	17.0	22.5	30	0.169	0.954	0.954	9.91	3.30	5.64	39.8
7	1	3	1	2	1	3	2	3	N/A	N/A	3	20	0.1	80	3×10^{-7}	10	13.7	27.1	41.9	54.4	30	0.411	5.64	5.64	910	303	13.7	27.9
8	1	3	2	3	2	1	3	1	N/A	N/A	5	100	0.3	60	1×10^{-6}	2.5	18.9	37.6	56.8	75.8	30	0.568	10.7	10.7	62.3	20.8	18.9	42.4
9	1	3	3	1	3	2	1	2	N/A	N/A	7	10	1	72.3	1×10^{-8}	5	2.55	5.61	7.66	10.4	30	0.078	0.205	0.205	187	62.2	2.61	20.4
10	2	1	1	3	3	2	2	1	N/A	N/A	3	100	1	72.3	3×10^{-7}	2.5	17.8	35.8	53.7	71.1	30	0.535	9.54	9.54	102	34.1	17.8	39.7
11	2	1	2	1	1	3	3	2	N/A	N/A	5	10	0.1	80	1×10^{-6}	5	64.1	127	190	253	30	1.90	120	120	1268	423	63.3	39.8
12	2	1	3	2	2	1	1	3	N/A	N/A	7	20	0.3	60	1×10^{-8}	10	44.1	88.4	132	177	30	1.33	58.5	58.5	583	194	44.2	40.0
13	2	2	1	2	3	1	3	2	N/A	N/A	3	20	1	60	1×10^{-6}	5	23.2	45.5	68.6	91.0	30	0.684	15.6	15.6	267	89.1	22.8	37.7
14	2	2	2	3	1	2	1	3	N/A	N/A	5	100	0.1	72.3	1×10^{-8}	10	8.56	17.1	25.4	34.0	30	0.255	2.17	2.17	16.7	5.57	8.50	41.1
15	2	2	3	1	2	3	2	1	N/A	N/A	7	10	0.3	80	3×10^{-7}	2.5	6.47	13.2	19.9	26.1	30	0.197	1.29	1.29	77.4	25.8	6.56	32.2
16	2	3	1	3	2	3	1	2	N/A	N/A	3	100	0.3	80	1×10^{-8}	5	8.32	16.2	25.1	33.3	30	0.249	2.07	2.07	194	64.5	8.31	30.3
17	2	3	2	1	3	1	2	3	N/A	N/A	5	10	1	60	3×10^{-7}	10	16.4	32.6	49.4	65.6	30	0.492	8.08	8.08	66.1	22.0	16.4	40.9
18	2	3	3	2	1	2	3	1	N/A	N/A	7	20	0.1	72.3	1×10^{-6}	2.5	40.9	81.4	122	162	30	1.22	49.4	49.4	257	85.6	40.6	42.8

N_A and N_B denote blank columns with no noise factors assigned.
