# Supplementary material for: Application of the Taguchi method to explore a robust condition of tumor-treating field treatment
Source: PLoS One. 2022 Jan 21;17(1):e0262133. doi: 10.1371/journal.pone.0262133 (PMC8782397; doi:10.1371/journal.pone.0262133)
Supplement: S3 Table — (PDF) [file pone.0262133.s004.pdf]

Anal. No.	Control factor										Frequency, f_{ac} (kHz)	Direction of the applied electric field, θ (deg)	Electrical conductivity of the extracellular medium, σ_e (S/m)	Relative permittivity of the extracellular medium, ε_e (-)			Output: Square root of mean $\lvert\nabla(E_{rms})^2\rvert$ in ROI								Sum of squared inputs	Sum of products of input and output	Sum of squared outputs (Total variation)	Variation of proportional term	Variation of noise	Variance of noise	Slope	S/N ratio
	C_A	C_B	C_1	C_2	C_3	C_4	C_G	C_H	C_A	C_B	C_1	C_2	C_3	C_4	C_G	C_H	Input: M_1		M_2		M_3		M_4		$r=\sum_{i=1}^{4} M_i^2$	$\sum My=\sum_{i=1}^{4} M_i y_i$	$S_T=\sum_{i=1}^{4} y_i^2$	$S_\beta=\frac{1}{r}\left(\sum My\right)^2$	$S_{Noise}=S_T-S_\beta$	$\sigma_{Noise}^2=V_{Noise}=\frac{S_{Noise}}{n-1}$	$\beta=\frac{1}{r}\sum My$	$S/N=\eta_{db}=10log\frac{\beta^2}{\sigma_{Noise}^2}=10log\frac{\beta^2}{V_{Noise}}$
																	N_{min}	N_{max}	N_{min}	N_{max}	N_{min}	N_{max}	N_{min}	N_{max}								
1	1	1	1	1	1	1	1	1	N/A	N/A	100	10	0.3	60	N/A	N/A	10.0×10^3	53.3×10^3	20.4×10^3	111×10^3	31.1×10^3	169×10^3	40.5×10^3	221×10^3	60	1.97×10^6	95.5×10^9	64.7×10^9	30.8×10^9	4.40×10^9	32.8×10^3	-6.10
2	1	1	2	2	2	2	2	2	N/A	N/A	200	0	1.2	72.3	N/A	N/A	16.7	44.2	33.1	84.8	49.7	130	69.2	172	60	1.80	64.2	54.0	10.2	1.46	30.0	-2.09
3	1	1	3	3	3	3	3	3	N/A	N/A	400	20	3	80	N/A	N/A	17.2	21.2	37.3	42.8	55.6	64.1	74.3	81.0	60	1.18	23.3	23.2	0.0867	0.0124	19.6	14.9
4	1	2	1	1	2	2	3	3	N/A	N/A	100	10	1.2	72.3	N/A	N/A	10.9	61.3	21.8	122	32.2	186	42.6	236	60	2.13	113	75.7	36.9	5.28	35.5	-6.22
5	1	2	2	2	3	3	1	1	N/A	N/A	200	0	3	80	N/A	N/A	17.8	44.4	36.0	87.4	54.5	128	73.9	169	60	1.83	64.6	55.7	8.90	1.27	30.5	-1.37
6	1	2	3	3	1	1	2	2	N/A	N/A	400	20	0.3	60	N/A	N/A	11.7	22.4	24.2	43.0	36.4	66.7	48.7	87.2	60	1.02	18.8	17.4	1.44	0.205	17.0	1.50
7	1	3	1	2	1	3	2	3	N/A	N/A	100	0	0.3	80	N/A	N/A	10.1	56.8	21.0	107	31.4	169	42.2	219	60	1.97	94.6	64.6	29.9	4.27	32.8	-5.98
8	1	3	2	3	2	1	3	1	N/A	N/A	200	20	1.2	60	N/A	N/A	15.4	40.4	32.4	81.6	48.3	126	63.0	164	60	1.71	58.6	49.0	9.64	1.38	28.6	-2.27
9	1	3	3	1	3	2	1	2	N/A	N/A	400	10	3	72.3	N/A	N/A	18.4	18.8	38.3	41.3	58.0	58.2	77.1	80.2	60	1.17	23.0	23.0	0.0133	0.00189	19.6	23.1
10	2	1	1	3	3	2	2	1	N/A	N/A	100	20	3	72.3	N/A	N/A	10.5	57.0	21.4	113	30.6	168	41.1	236	60	2.04	103	69.4	33.7	4.82	34.0	-6.20
11	2	1	2	1	1	3	3	2	N/A	N/A	200	10	0.3	80	N/A	N/A	13.0	41.2	26.8	84.5	40.3	122	51.3	166	60	1.63	56.2	44.3	11.9	1.70	27.2	-3.62
12	2	1	3	2	2	1	1	3	N/A	N/A	400	0	1.2	60	N/A	N/A	17.2	20.5	34.6	37.5	51.9	59.7	69.4	77.3	60	1.10	20.4	20.3	0.0740	0.0106	18.4	15.1
13	2	2	1	2	3	1	3	2	N/A	N/A	100	0	3	60	N/A	N/A	11.0	56.2	22.5	126	32.5	174	43.9	256	60	2.18	118	79.5	39.0	5.57	36.4	-6.24
14	2	2	2	3	1	2	1	3	N/A	N/A	200	20	0.3	72.3	N/A	N/A	12.4	39.0	25.6	80.0	38.8	118	51.5	162	60	1.59	53.1	42.0	11.1	1.58	26.5	-3.55
15	2	2	3	1	2	3	2	1	N/A	N/A	400	10	1.2	80	N/A	N/A	17.1	20.8	34.8	42.1	52.0	60.7	71.4	80.2	60	1.14	21.6	21.5	0.111	0.0158	18.9	13.6
16	2	3	1	3	2	3	1	2	N/A	N/A	100	20	1.2	80	N/A	N/A	10.4	55.5	21.1	114	30.6	167	41.1	231	60	2.02	101	68.0	32.8	4.68	33.7	-6.16
17	2	3	2	1	3	1	2	3	N/A	N/A	200	10	3	60	N/A	N/A	17.5	42.2	35.7	90.0	53.4	130	71.5	163	60	1.80	62.7	53.8	8.86	1.27	29.9	-1.50
18	2	3	3	2	1	2	3	1	N/A	N/A	400	0	0.3	72.3	N/A	N/A	13.1	21.8	25.2	43.1	38.9	67.2	51.4	89.0	60	1.05	19.7	18.4	1.30	0.186	17.5	2.17

C_A, C_B, C_G, and C_H denote blank columns with no control factors assigned.
